# An update of KAIKObase, the silkworm genome database

**DOI:** 10.1093/database/baaa099

**Published:** 2021-02-27

**Authors:** Ching-chia Yang, Kakeru Yokoi, Kimiko Yamamoto, Akiya Jouraku

**Affiliations:** Institute of Agrobiological Sciences, National Agriculture and Food Research Organization, 1-2 Owashi, Tsukuba, Ibaraki 305-8634, Japan; Institute of Agrobiological Sciences, National Agriculture and Food Research Organization, 1-2 Owashi, Tsukuba, Ibaraki 305-8634, Japan; Institute of Agrobiological Sciences, National Agriculture and Food Research Organization, 1-2 Owashi, Tsukuba, Ibaraki 305-8634, Japan; Institute of Agrobiological Sciences, National Agriculture and Food Research Organization, 1-2 Owashi, Tsukuba, Ibaraki 305-8634, Japan

## Abstract

KAIKObase was established in 2009 as the genome database of the domesticated silkworm *Bombyx mori*. It provides several gene sets and genetic maps as well as genome annotation obtained from the sequencing project of the International Silkworm Genome Consortium in 2008. KAIKObase has been used widely for silkworm and insect studies even though there are some erroneous predicted genes due to misassembly and gaps in the genome. In 2019, we released a new silkworm genome assembly, showing improvements in gap closure and covering more and longer gene models. Therefore, there is a need to include new genome and new gene models to KAIKObase. In this article, we present the updated contents of KAIKObase and the methods to generate, integrate and analyze the data sets.

**Database URL**: https://kaikobase.dna.affrc.go.jp

## Introduction

*Bombyx mori* L., the domesticated silkworm, has been living with human beings for thousands of years, forming sericulture industry and providing us materials for clothes and artwork. It has lost its ability to move, fly and forage during the domestication process, making it an ideal experimental animal in the laboratory. As a consequence, for a long time, silkworm has been widely studied for revealing genetic mechanisms of insect physiology (1–5) as a model organism. It is also a useful reference and platform for studying lepidopteran pests. Lepidopteran pests cause great damages to crops and vegetables and those developing pesticide resistance become acute problems. Therefore, there is an urge need to reveal the mechanism of pesticide resistance for pest monitoring and management. With the help of silkworm, genetic basis of resistance to several pesticides has been identified in other lepidopteran species (6–9) and molecular diagnosis method could be developed based on the discovery (10). Moreover, in recent years, its ability in producing bulk silk proteins in cocoons makes it an ideal protein factory to produce proteins of interests with low prices through genetic engineering. Transgenic silkworm has been applied widely, including the production of antibodies, drugs and cosmetic materials such as collagen (11, 12). The silk itself has also attracted a great attention as a material for diverse biomedical usages (13–16). Precise genomic and genetic information will thus be needed for making full usages of silkworm with genetic engineering technologies. As a consequence, a database of silk genomic resource not only can be the basis of silkworm studies but also facilitates researches of diverse fields.

The high-quality genome sequences and genetic maps of silkworm were first released independently by a Japanese group and a Chinese group in 2004 (17, 18) and then an upgraded version was released by the collaboration of these two groups in 2008 (19). KAIKObase (20) was then constructed based on the genome of 2008, providing a wide range of knowledge including physical and genetic linkage maps, as well as gene structures and annotations. It also provides services for keyword, position and similar sequence for the researchers of entomology, pest management, biomaterials and so on. Since the launch in 2009, KAIKObase keeps a steady and indispensable knowledge base of silkworm genetics for researchers with almost 1 million access per year.

Since it started to provide services, KAIKObase has been updated several times, from integrating other silkworm-related resources (version 2), changing to Chado database (version 3.0.0), adding full-length cDNA sequence data set (21) (version 3.1.0), to the current update in 2013 (version 3.2.2) of adding gene description pages including sequences, expression, automated functional annotation and orthologous genes of related insect and pest species. However, of all these versions of KAIKObase, the genome assembly was not updated. Finally, in 2019, an improved genome assembly generated using PacBio long reads and Illumina short reads was released (22). Many gaps were closed in the new genome, and more genes with longer average length were predicted in the new genome than in the genome of 2008 (16 880 genes of 1551 bp on average in the new genome versus 14 623 genes of 1224 bp on average in genome of 2008). Therefore, there is a need to update KAIKObase using the latest information of genome and genes. In addition, information used for gene annotation is out of date because there was no update for annotation since its last update in 2013. In the past several years, databases that are widely used for functional annotation of genes, such as National Center for Biotechnology Information (NCBI) non-redundant (nr) protein database (23), InterPro (24) and Gene Ontology (GO) (25), had been updated frequently, and the amount of data has increased tremendously. As a result, annotating genes using the latest genetic information is also needed for the genes in KAIKObase.

For the latest KAIKObase (version 4) introduced here, we used the improved silkworm genome as the reference genome. Gene structure of newly predicted protein-coding genes (hereafter, new gene models) and gene contigs assembled from the transcriptome data (26) (hereafter, reference transcripts) are visible from the genome browser. Detailed annotations are available for each predicted gene. We updated the sequence search service by adding more gene sequences (new gene models and reference transcripts) to search against. We also manually curated genes related to detoxification and target genes of pesticides, as well as genes related to silk production, aiming to provide accurate gene information to the users. Throughout these updates, we anticipate that KAIKObase will still be an irreplaceable database of silkworm genome and genetics in the next decades.

## Overview of the update

**Figure 1.**
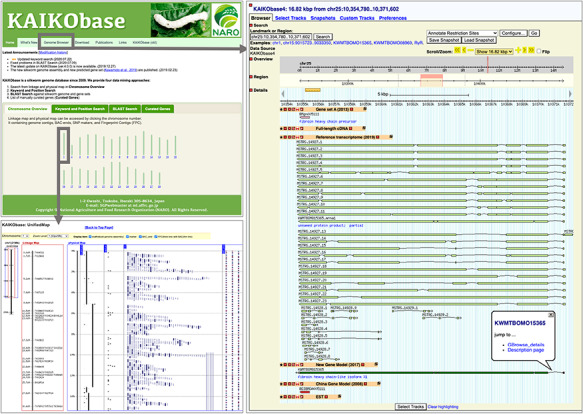
Webpages of KAIKObase. Top left: the homepage of KAIKObase, where genetic maps, keyword and sequence search, lists of curated genes, as well as the link to the genome browser, are available; bottom left: genetic map with markers and BAC sequences; right: the page of genome browser with an example of predicted, curated and transcribed sequences from fibroin heavy chain.

**Figure 2.**
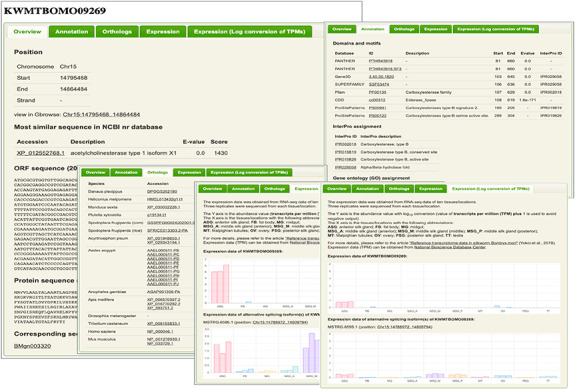
An example of description page. The information of chromosomal positions, nucleotide and amino acid sequences, functional annotations, orthologs and expression patterns [original transcripts per million (TPM) values and log_10_ conversion of TPM values with an offset of 1] are shown.

Main updates of KAIKObase (version 4) include that (i) new genome assembly is used as the reference genome in the genome browser Gbrowse (27); (ii) 16 880 new gene models and 51 926 reference transcripts are accessible in the genome browser, while previous gene sets (Gene set A (21), old China gene models (19), etc.) are still available (as shown in Figure 1 (right-hand side)); (iii) sequence search by BLAST (28) allows users to search against not only the new genome sequence but also the new gene models and reference transcripts; (iv) users are able to search genes based on their functional annotations for new data sets (new gene models and reference transcripts) as well as previous data sets (Gene set A, old China gene models and full-length cDNAs) in keyword search; (v) functional annotations of each new gene model and its expression patterns in various tissues, as well as orthologs of closely related lepidopteran species and model vertebrate and insect organisms, are available in a webpage called ‘description page’ (Figure 2) and (vi) previously identified single-nucleotide polymorphism (SNP) markers in an existing linkage map, bacterial artificial chromosome (BAC)-end sequences and fingerprint contigs (FPCs) (29) were mapped onto the new genome assembly (Figure 1, bottom left).

In addition to the above updates, we also provide a list of manually curated genes because they are frequently investigated. Among these genes, 246 are related to detoxification [52 ATP-binding-cassette (ABC) transporters, 87 carboxylesterases (COEs), 23 glutathione S-transferases (GSTs) and 84 cytochrome P450 genes (CYPs)], which were already curated (22), while 16 target genes of pesticides and 7 genes related to silk production were curated here (Table 1 and Supplementary Table 1).

**Table 1.** List of manually curated genes of pesticide targets and silk proteins.

Curated gene	Name	Predicted gene model
Target genes of pesticide		
BmTargetGene-01	ATP-binding cassette transporter A2 (ABCA2)	KWMTBOMO10323M
BmTargetGene-02	ATP-binding cassette transporter B1 (ABCB1)	KWMTBOMO09033
BmTargetGene-03	ATP-binding cassette transporter C2 (ABCC2)	KWMTBOMO08969
BmTargetGene-04	AChE1	KWMTBOMO09269
BmTargetGene-05	Chitin synthase 1	KWMTBOMO02041M
BmTargetGene-06	Ecdysone receptor	KWMTBOMO05605
BmTargetGene-07	Gamma aminobutyric acid receptor	KWMTBOMO00230M
BmTargetGene-08	Glutamate-gated chloride channel	KWMTBOMO04162M
BmTargetGene-09	Nicotinic acetylcholine receptor (nAChR) α6	KWMTBOMO03109M
BmTargetGene-10	nAChR β1	KWMTBOMO02752M
BmTargetGene-11	Ryanodine receptor	KWMTBOMO14367-14 368-14371M
BmTargetGene-12	Succinate dehydrogenase A (SdhA)	KWMTBOMO01346
BmTargetGene-13	SdhA-like (SdhA-like)	KWMTBOMO00596-00597M
BmTargetGene-14	SdhB (SdhB)	KWMTBOMO15500
BmTargetGene-15	SdhB-like	KWMTBOMO08409
BmTargetGene-16	Voltage-gated sodium channel (Nav channel)	KWMTBOMO12624-12625M
Fibroin		
BmFibroin-01	Light chain	KWMTBOMO08464
BmFibroin-02	Heavy chain	KWMTBOMO15365M
BmFibroin-03	P25	KWMTBOMO01001
Sericin		
BmSericin-01	Sericin 1B	KWMTBOMO06216M
BmSericin-02	Sericin 2	KWMTBOMO06334M
BmSericin-03	Sericin 3	KWMTBOMO06311M
BmSericin-04	Sericin 4	KWMTBOMO06324-06325-06326M

The gene is curated from the predicted gene model if the predicted gene model ends with an ‘M’ as ‘modified’

We prepared several downloadable files for the users, including the new genome assembly sequences and sequences of new gene models that are also available in SilkBase (http://silkbase.ab.a.u-tokyo.ac.jp), the correspondence between new gene models and Gene set A, and functional annotation of the new gene models and curated genes. All of these files are available through the following URL: https://kaikobase.dna.affrc.go.jp/KAIKObase_download.html.

## Genetic markers on new genome

To reflect the chromosomal positions of SNP markers, BAC-end sequences and FPCs on the new genome assembly, we mapped these sequences using BLASTN (version 2.2.30+) with threshold e-value of 1e-200 (for SNP markers and FPCs) and 0.1 (for BAC-end sequences). An SNP marker or FPC is successfully mapped if the query sequence can be found in only one chromosomal position and the aligned region covers more than 80% of the query sequence. All of the 1532 SNP markers were mapped onto the new genome assembly, while 4726 of 4754 FPCs (99.4%) could be mapped. The BAC-end sequences are considered to be successfully mapped if the aligned region covers more than 50% of the query sequence. Approximately, 97% of the BAC-end sequences (133 242 out of 137 219) could be mapped onto the new genome assembly.

## Description page of new gene models

We created ‘description pages’ (Figure 2) for 16 880 new gene models to provide their detailed information, including chromosomal positions, nucleotide and amino acid sequences, corresponding gene accession(s) in the previous KAIKObase, assignment of domains and motifs, orthologous genes in closely related insects including famous lepidopteran pests and expression patterns. The methods and results for generating information are introduced as follows.

### Corresponding gene accession in the previous KAIKObase

As the gene accessions are different from those in old KAIKObase, it is necessary for the users to know the correspondence between new and old gene accessions. To retrieve the same genes from new and old KAIKObase, we first performed a BLASTP search (version 2.6.0+) (28) for the new gene models against the recent gene set in old KAIKObase (Gene set A (21)) with default parameters. Next, we mapped the old gene models to the new genome assembly by minimap2 (version 2.17) (30). Combining these two results, we considered both an old gene model and a new gene model are the same gene when: (i) the old gene model is the hit of the new gene model and (ii) they can be mapped to the same chromosomal position. Under these criteria, 13 304 of 16 880 new gene models could be assigned by 15 810 old gene models. Among the 3576 new gene models that no corresponding old gene model could be assigned, 1912 were mapped onto the different chromosomal positions compared with their Gene set A hits. The remaining 1664 new gene models show no hit to old genes, of which more than half are most similar to genes identified in other species. These genes may not be identified in the previous silkworm genome analysis or silkworm studies and could be candidates for further investigation. URL links from the description page of old gene models were added into the new description page so that the users can transfer between new and old gene accessions.

### Domain and motif assignment

We used InterProScan (version 5.38–76.0) (31) to assign domains and motifs to the new gene models using the following databases: Conserved Domain Database, Gene3D, Pfam, PIRSF, PANTHER, PROSITE (both patterns and profiles), Simple Modular Architecture Research Tool, SUPERFAMILY and TIGRFAMs. With the default parameter settings, 2312 new gene models were not assigned any InterProScan result. For the rest of the gene models, the position and e-value of each domain or motif are listed in their description pages. Accession numbers of InterPro and GO and their descriptions are also listed in the description pages.

### Orthologous genes in other lepidopterans and model organisms

We provide a list of orthologous genes of each new gene model in closely related lepidopterans [*Danaus plexippus* (32), *Heliconius melpomene* (33), *Manduca sexta* (34), *Plutella xylostella* (33) and *Spodoptera frugiperda* (both corn and rice ecotypes) (35)], non-lepidopteran model insects [*Acyrthosiphon pisum* (36), *Aedes aegypti* (37), *Anopheles gambiae* (37), *Apis mellifera* (38), *Drosophila melanogaster* (39) and *Tribolium castaneum* (40)], human (41) and mouse (42) (Table 2). The orthologs were identified by OrthoFinder (version 2.3.3) (43) with the default setting of parameters. Among the 16 880 silkworm genes, 65.1–68.3% are orthologous to lepidopteran genes, 43.0–48.5% are orthologous to non-lepidopteran insect genes, while 37.6% and 37.3% are orthologous to human and mouse genes, respectively. Further analysis showed that 3748 genes (22.2%) are orthologous among all the species used here, while 4484 (26.6%) and 8000 (47.4%) are orthologous among all the insects and lepidopterans, respectively.

**Table 2.** Number of genes in silkworm that have orthologs in model organisms of vertebrates and insects

Species	Number of silkworm ortholog	Accession number/version^a^
Lepidopteran insects		
*Danaus plexippus* (monarch butterfly)	11 054 (65.5%)	OGS2.0 (MonarchBase)
*Heliconius melpomene* (postman butterfly)	11 345 (67.2%)	Hmel2.5 (LepBase)
*Manduca sexta* (tobacco hornworm)	11 446 (67.8%)	GCF_000262585.1 (NCBI)
*Plutella xylostella* (diamondback moth)	10 982 (65.1%)	pacbiov1 (LepBase)
*Spodoptera frugiperda* (fall armyworm) (corn ecotype)	11 289 (66.9%)	OGS2.2 (LepidoDB)
*Spodoptera frugiperda* (fall armyworm) (rice ecotype)	11 525 (68.3%)	OGS2.3 (LepidoDB)
Non-lepidopteran insects		
*Acyrthosiphon pisum* (pea aphid)	7251 (43.0%)	GCF_005508785.1 (NCBI)
*Aedes aegypti* (yellow fever mosquito)	7972 (47.2%)	AaegL5.2 (VectorBase)
*Anopheles gambiae* (African malaria mosquito)	7743 (45.9%)	AgamP4.12 (VectorBase)
*Apis mellifera* (western honeybee)	7706 (45.7%)	GCF_003254395.2 (NCBI)
*Drosophila melanogaster* (fruit fly)	7625 (45.2%)	Dmel Release 6.29 (FlyBase)
*Tribolium castaneum* (red flour beetle)	8192 (48.5%)	GCF_000002335.3 (NCBI)
Vertebrates		
*Homo sapiens* (human)	6348 (37.6%)	GRCh38 (NCBI)
*Mus musculus* (mouse)	6293 (37.3%)	GRCm38.p6 (NCBI)

^a^ the gene set was obtained from the database written in the parenthesis.

### Expression pattern

The transcriptome data (26) include mRNAs extracted from the fat body, midgut, Malpighian tubules, testis, anterior silk gland, anterior, middle and posterior parts of the middle silk gland, and posterior silk gland of one male P50T larva and from the ovary of a female larva. The expression level was calculated as transcripts per million (TPM). Splicing alternatives of a new gene model are transcripts whose: (i) splicing patterns are different from that of the new gene model and (ii) exon regions overlapped with any part of those of the new gene model. The transcriptome data covered 98.3% of the 16 880 new gene models, among which 9617 contain 28 526 splicing alternatives in total. The expression patterns of its splicing alternatives are also shown in the description page of the new gene model. We also provide log_10_ conversion of TPM values with an offset of 1 (for avoiding negative values) for users to make intuitive comparisons easier.

## Manually curated gene families

We curated several predicted gene models manually to provide verified intron–exon structures and sequences for our users. The curation was focused on genes related to pesticide resistance and silk production which have drawn much attention for their applications in wide ranges of fields. We collected the complete coding sequences of 16 target genes of pesticides and 6 genes related to silk production (fibroin and sericin) from NCBI nr nucleotide database for gene curation. Exonerate (version 2.2.0) (44) was used to align the complete coding sequences onto the genome assembly to identify the correct gene models of these sequences by the alignment model of est2genome. Nine of the 16 target genes, all of the 3 genes from sericin and 1 of the gene from fibroin were mapped onto the genome as different gene models from the predicted ones (Table 1). Three target genes of pesticides, BmTargetGene-01, BmTargetGene-02 and BmTargetGene-03, were mapped onto the same positions as curated ABC transporters BmABC-39, BmABC-34 and BmABC-30, respectively. We also retrieved intron–exon structure of a newly identified sericin protein (sericin 4) from the work of Dong *et al*. (45) and compared it to the reference transcript MSTRG.2610.1 for the curation of sericin 4. These curated genes are accessible in the genome browser in an independent track, and their description pages were created, containing the positional information, sequences, functional annotations and orthologous genes as the description pages of new gene models except for the information of expression patterns. The method to identify orthologous genes in other species is the same as mentioned above.

We further investigated orthologs of four detoxification-related gene families in different species (Supplementary Tables 1 and 2). ABC transporters are ubiquitous across the all the phyla and are fundamental to import essential nutrients and export toxins (46, 47), as our data showed they are highly conserved among all of the lepidopteran species and even among insects and animals. COEs, GSTs and CYPs are less conserved and relatively species specific between different lepidopterans from our data, which may indicate that they could contribute to the detoxification of different targets in each species as they are well-known to be involved in detoxifying a wide range of xenobiotics (48–51). The 16 target genes of pesticides are relatively conserved among all the species compared with other genes (Supplementary Table 2), since most of the target genes possess essential functions for the cells as transporters, receptors or channels. Although these genes are conserved between insects and human, and the effects of pesticides are generally less toxic to mammals than insects because of their specificity to insects (52, 53).

Sericin and fibroin are essential for silkworm in silk production, being the coat and the core of the silk, respectively. Orthologs of sericin proteins could not be identified in other species, showing that these proteins are very specific in silkworm. On the other hand, orthologs of silkworm fibroin proteins can be found in other lepidopteran species (Supplementary Table 2). Fibroin P25 protein is orthologous among silkworm and all of the six lepidopteran species, while light-chain fibroin (L-fibroin) are missing in both ecotypes of *S. frugiperda* and heavy-chain fibroin (H-fibroin) are missing in *D. plexippus* and corn ecotype of *S. frugiperda* (Supplementary Table 1). However, we searched MonarchBase (32) and LepidoDB (http://bipaa.genouest.org/is/lepidodb/spodoptera_frugiperda/) and found that there are genes annotated as H-fibroin in *D. plexippus* (http://monarchbase.umassmed.edu/tools3/Get_gene.cgi?id=DPOGS204188) and *S. frugiperda* (several genes such as GSSPFG00007524001-PA, SFRURICE0000006288-PA, etc. can be accessed from the search form at https://bipaa.genouest.org/sp/spodoptera_frugiperda_pub/ with keyword of ‘fibroin heavy chain’). Since the property of the silk is largely determined by H-fibroin (54), *D. plexippus* and *S. frugiperda* may have different silk property from silkworm. We also noticed that *S. frugiperda* harbors L-fibroin which has homologs in another *Spodoptera* species*, Spodoptera. litura*, again suggesting its different silk property. It may be worthy to investigate the fibroin proteins in *D. plexippus* and *S. frugiperda* for the search of new silk materials.

## Conclusion and future perspectives

KAIKObase has supported researches of silkworm and insects since 2009 and is now providing the latest genetic and genomic information for the scientific community. The new genome and gene models showed more accurate sequences and gene sets than old ones, and the genes were annotated with the latest information. The updated KAIKObase will continue to contribute to the researches of silkworm and insects, as well as the sericulture industry and biotechnological applications.

The decreasing cost of sequencing makes it easy to collect large-scale sequence data in a short time. Therefore, it is expected that high-quality genome assemblies of various silkworm lineages will be determined to broaden our knowledge of silkworm. Meanwhile, the use of genetic markers obtained using next-generation sequencing such as restriction-site associated DNA sequencing and genotyping by sequencing becomes a popular and preferable method in a wide range of fields, including population genotyping, quantitative trait loci (QTL)-mapping and breeding (55). Our future objectives for updating KAIKObase will include collecting more silkworm genomes and genetic markers as population-level data to keep it as a comprehensive and indispensable repository for silkworm research.

## Supplementary Material

baaa099_Supp
