# Supplementary material for: An update of KAIKObase, the silkworm genome database
Source: Database (Oxford). 2021 Feb 27;2021:baaa099. doi: 10.1093/database/baaa099 (PMC7918157; doi:10.1093/database/baaa099)
Supplement: baaa099_Supp [file baaa099_supp.zip › SupplementaryTable2.docx]

**Supplementary Table 2.** Number of detoxification-related genes, target genes of pesticides, and silk production-related genes that are orthologous to silkworm genes in model organisms of vertebrates and insects. Number in each parenthesis in the header is the number of gene in each category in silkworm, and number in each parenthesis inside the table is the number of orthologs in silkworm. (ABC: ABC transporter; COE: carboxylesterase; GST: glutathione S-transferase; CYP: cytochrome P450)

| **Species** | **ABC (52)** | **COE (87)** | **GST (23)** | **CYP (84)** | **Target gene (16)** | **Fibroin (3)** | **Sericin (4)** |
| --- | --- | --- | --- | --- | --- | --- | --- |
| **Lepidopteran insects** |  |  |  |  |  |  |  |
| *D. plexippus* | 55 (50) | 47 (57) | 18 (18) | 53 (52) | 20 (16) | 2 (2) | 0 |
| *H. melpomene* | 49 (49) | 49 (59) | 18 (17) | 60 (54) | 18 (16) | 3 (3) | 0 |
| *M. sexta* | 58 (50) | 84 (70) | 35 (22) | 67 (69) | 17 (16) | 3 (3) | 0 |
| *P. xylostella* | 50 (49) | 47 (66) | 17 (20) | 46 (52) | 19 (16) | 4 (3) | 0 |
| *S. frugiperda* (corn ecotype) | 56 (51) | 79 (71) | 20 (21) | 63 (56) | 16 (16) | 1 (1) | 0 |
| *S. frugiperda* (rice ecotype) | 56 (50) | 89 (59) | 32 (20) | 90 (52) | 15 (16) | 2 (2) | 0 |
| Shared among Lepidopteran | (47) | (36) | (14) | (34) | (16) | (1) | 0 |
| **Non-lepidopteran insects** |  |  |  |  |  |  |  |
| *A. pisum* | 51 (39) | 17 (14) | 10 (7) | 46 (31) | 21 (14) | 0 | 0 |
| *A. aegypti* | 59 (41) | 40 (19) | 13 (12) | 40 (39) | 22 (13) | 0 | 0 |
| *A. gambiae* | 36 (38) | 16 (18) | 15 (12) | 51 (37) | 13 (13) | 0 | 0 |
| *A. mellifera* | 60 (37) | 26 (17) | 11 (9) | 51 (30) | 20 (13) | 0 | 0 |
| *D. melanogaster* | 72 (43) | 32 (16) | 22 (12) | 35 (40) | 16 (13) | 0 | 0 |
| *T. castaneum* | 43 (37) | 23 (20) | 14 (12) | 61 (45) | 19 (14) | 0 | 0 |
| Shared among insects | (28) | (10) | (6) | (12) | (12) | 0 | 0 |
| **Vertebrates** |  |  |  |  |  |  |  |
| *H. sapiens* (human) | 47 (27) | 54 (13) | 22 (8) | 21 (10) | 44 (13) | 0 | 0 |
| *M. musculus* (mouse) | 30 (26) | 40 (17) | 14 (9) | 25 (17) | 22 (13) | 0 | 0 |
| Shared among all species | (22) | (7) | (3) | (1) | (11) | 0 | 0 |
